# Can Presurgical Interhemispheric EEG Connectivity Predict Outcome in Hemispheric Surgery? A Brain Machine Learning Approach

**DOI:** 10.3390/brainsci13010071

**Published:** 2022-12-30

**Authors:** Chiara Pepi, Mattia Mercier, Giusy Carfì Pavia, Alessandro de Benedictis, Federico Vigevano, Maria Camilla Rossi-Espagnet, Giovanni Falcicchio, Carlo Efisio Marras, Nicola Specchio, Luca de Palma

**Affiliations:** 1 Rare and Complex Epilepsies Unit, Department of Neuroscience, Bambino Gesù Children’s Hospital, IRCCS, Full Member of European Reference Network EpiCARE, 00165 Rome, Italy; 2 Neurosurgery Unit, Bambino Gesù Children’s Hospital, IRCCS, Full Member of European Reference Network EpiCARE, 00165 Rome, Italy; 3 Neurology Unit, Bambino Gesù Children’s Hospital, IRCCS, Full Member of European Reference Network EpiCARE, 00165 Rome, Italy; 4 Neuroradiology Unit, Imaging Department, Bambino Gesù Children’s Hospital, 00165 Rome, Italy; 5 Department of Basic Medical Sciences, Neurosciences and Sense Organs, University of Bari Aldo Moro, 70121 Bari, Italy

**Keywords:** hemispherotomy, seizure prediction, outcome, brain machine learning

## Abstract

Objectives: Hemispherotomy (HT) is a surgical option for treatment of drug-resistant seizures due to hemispheric structural lesions. Factors affecting seizure outcome have not been fully clarified. In our study, we used a brain Machine Learning (ML) approach to evaluate the possible role of Inter-hemispheric EEG Connectivity (IC) in predicting post-surgical seizure outcome. Methods: We collected 21 pediatric patients with drug-resistant epilepsy; who underwent HT in our center from 2009 to 2020; with a follow-up of at least two years. We selected 5-s windows of wakefulness and sleep pre-surgical EEG and we trained Artificial Neuronal Network (ANN) to estimate epilepsy outcome. We extracted EEG features as input data and selected the ANN with best accuracy. Results: Among 21 patients, 15 (71%) were seizure and drug-free at last follow-up. ANN showed 73.3% of accuracy, with 85% of seizure free and 40% of non-seizure free patients appropriately classified. Conclusions: The accuracy level that we reached supports the hypothesis that pre-surgical EEG features may have the potential to predict epilepsy outcome after HT. Significance: The role of pre-surgical EEG data in influencing seizure outcome after HT is still debated. We proposed a computational predictive model, with an ML approach, with a high accuracy level.

## 1. Introduction

Hemispherotomy (HT) is a surgical option to treat refractory seizures due to hemispheric structural lesions. The purpose of HT is to functionally isolate the epileptogenic zone, which is widely diffused throughout the hemisphere [1]. Seizures may arise from different regions within the same hemisphere, revealing an intrinsic multifocality [2].

Epilepsies associated with hemispheric structural lesions can be seen in the context of specific diseases, such as Sturge–Weber syndrome (SWS), neurocutaneous diseases [3], Rasmussen syndrome, hemiplegia-hemiconvulsion-epilepsy syndrome (HHE), isolated hemispheric malformations of cortical development, vascular ischemic/hemorrhagic event, trauma or infectious events [4].

Patients with such lesions usually may manifest with hemiparesis, delayed neurocognitive development and drug-resistant epilepsy. Epilepsy surgery currently represents the most effective therapeutic option [5].

Hemispheric surgery is associated with high seizure freedom rates, ranging from 50% to 90% [5,6,7,8,9,10], and relatively low complication rates in specialized centers [6,10]. The most significant predictive factor of worse outcome after HT is developmental etiology, including malformations of cortical development [3,11], whereas type of surgical procedure does not influence the outcome [12,13,14]. Beside incomplete hemispheric disconnection, it is not fully elucidated why some patients do have seizures persistence: the presence of bilateral pathologic findings [3,6,15], contralateral EEG [3,16,17,18] and neuroimaging abnormalities have been associated with a worse seizure outcome after surgery [6].

Despite normal contralateral MRI and complete disconnection on post-operative MRI, still a substantial subset of patients do not achieve seizure freedom [3]. It still remains unclear, which is the best combination of preoperative characteristics associated with best chance of seizure freedom following HT.

Recently, a clinical scoring to predict seizure freedom in children undergoing cerebral hemispheric surgery has been proposed [15]. It comprises presurgical data (age at seizure onset, generalized seizure semiology), underlying pathologic substrate (stroke vs. non-stroke etiology), presence of contralateral interictal FDG-PET hypometabolism, and history of previous resective surgery. However, the role of presurgical neurophysiologic data in influencing seizure outcome is still debated [3,6,16].

Interhemispheric Connectivity (IC) in EEG signals has been extensively analyzed in generalized epilepsies [19,20,21,22], vascular injuries [23] and in psychiatric disorders [24,25,26]. IC has never been studied as a factor influencing seizure outcome after hemispheric surgery, and we aimed to study this parameter through an automated method.

Machine Learning (ML) provides an opportunity to objectively manipulate multimodal data, allowing for the production of algorithms, and it therefore has been widely applied also for medical purposes [27]. ML techniques, including Artificial Neural Networks (ANNs), have been used in the field of epilepsy research for automated epilepsy diagnosis [28,29], seizure lateralization [30,31], and prediction of postsurgical seizure freedom [32,33,34,35,36].

In this study, we aimed to use ANN as computational ML technique to investigate IC. We compared selected pre-surgical EEG features extracted from both hemispheres, pursuing the hypothesis that an increased IC may be a predicting factor for seizure recurrence after surgery.

## 2. Methods

### 2.1. Patients

We retrospectively recruited all pediatric patients (<18 years) who underwent HT for drug-resistant epilepsy from January 2009 to April 2020, in Bambino Gesù Children Hospital, Rome, Italy. All patients in our center underwent vertical parasagittal HT, as previously published [12].

A total of 30 patients have been included in this study: we collected clinical, neuroradiological, and neurophysiological data.

We filtered patients according to the following inclusion criteria:

- Available pre-surgical clinical and EEG data during wakefulness and sleep;
- Electroclinic concordance: unilateral seizure onset concordant with side of the lesion at brain MR;
- Absence of signal or morphological abnormalities within the contralateral hemisphere on pre-surgical MR;
- Complete disconnection evaluated at post-surgical brain MR, after multidisciplinary re-evaluation;
- At least two years of post-surgical follow-up;
- No previous surgeries for the treatment of epilepsy.

Out of 30 patients extracted from our database, we included 21 patients in this study.

### 2.2. Presurgical Assessment

All patients underwent routine pre-surgical evaluation, including full clinical history and neurological examination, visual analysis of long-term video-EEG monitoring with multiple typical seizures recorded, and three Tesla brain MR with epilepsy protocol. Seizure were classified according to the ILAE Position Paper for Classification and Terminology [37]. Seizure outcome was recorded using the Engel classification [38]. Potential surgical candidates were discussed during multi-disciplinary epilepsy surgery meetings to determine suitability for surgery. The decision to offer surgery was based on predicted seizure outcome from pre-surgical data and surgical risks.

### 2.3. EEG Data Recording, Acquisition and Processing

**Figure 1:**
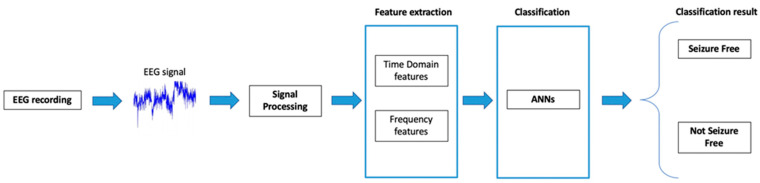
Diagram of the proposed surgery outcome prediction method of our study.

Our study work-up included four stages: EEG recordings and acquisition, signal processing, EEG features extraction and classification through Artificial Neural Network, as shown in Figure 1.

EEG recordings were obtained with a video-EEG monitoring system (Micromed, Treviso, Italy) at the Rare and Complex Epilepsy unit of the Bambino Gesù Children Hospital in Rome, Italy. The 10–20 electrode montage was used for scalp recordings. Monopolar recordings were obtained with a sampling frequency of 256 Hz, band-pass filtered at between 0.5 and 45 Hz (4th order Butterworth filter) and 16-bit resolution. The reference electrode was set on G2. The extraction of EEG data was performed primarily by two expert neurophysiologists (CP, GCP) through visual inspection.

Before filtering, EEG signals are retained to their initial raw format and two 60-s segments have been extracted for both wakefulness (resting state with eyes closed) and sleep (sleep stage II) for every patient, considering the most artifact and epileptic abnormalities free fragments. For each patient, a 120-s-length EEG was extracted.

The continuous long-term raw EEG data were first segmented into five-s non-overlapping windows [39]. No additional artifact suppression methods were employed.

### 2.4. EEG Features Extraction and Classification through Artificial Neural Network (ANN)

We used, as input of our ANN, a set of EEG features during wakefulness and sleep, from 5-s non-overlapping windows of 19 electrodes EEG recordings, removing midline electrodes.

To quantify EEG signal, we extracted these features, shown in Table 1:

- Power Spectral Density (PSD) [40], related to frequency domain;
- Hjorth parameters, including Mobility, Complexity and Activity [41], related to time domain, respectively.

Considering a dataset of 42 EEG segments (two segments for 21 patients), we extracted 9 EEG features (6 for PSD + 3 for Hjorth analysis) from each acquiring channel (16 electrodes) for a total of 144 features per EEG (9 × 16). Definitively, we used 288 EEG features for each patient.

We calculated mean values of each feature for each electrode, divided for “healthy” and “pathological” hemisphere and sub-sequentially we calculated differences between the two hemispheres for each feature.

We performed a feature selection, dividing our features in four different subsets, in order to reduce input data, prevent overfitting and study the best adaptation of our model. We used the following features datasets:

- Dataset 1: all features;
- Dataset 2: all features calculated only in the total band frequency [0.5–45 Hz];
- Dataset 3: frequency-domain features (PSD) for every signal band;
- Dataset 4: time-domain features (Hjorth parameters) for every band;
- The Mean Square Error (MSE) was calculated to select the most accurate training set.

We used the inter-hemispheric difference of connectivity metrics as input in ANN, implemented using MATLAB R2019a software package, to estimate epilepsy outcome after hemispherotomy (seizure free SF vs. non-seizure free NSF).

**Figure 2:**
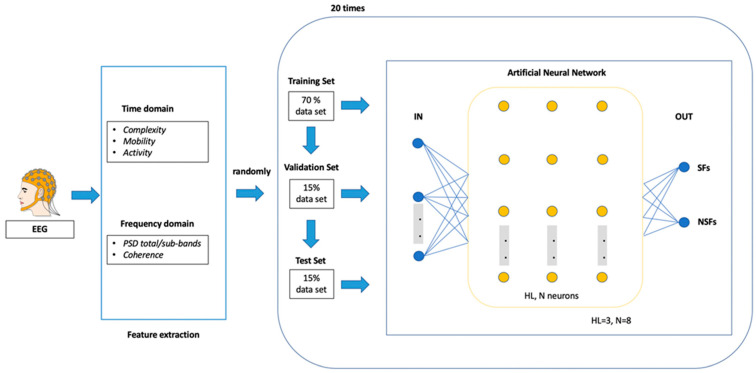
Artificial Neural Network (ANN) architecture of the model.

ANN architecture is shown in Figure 2.

We tested nine different network topologies as a predictive model. These architectures were implemented by varying the number of Hidden Layers (HL) and the number of Neurons (N) populating each HL.

The number of HL varied in the range 1–2, while the number N in each HL varied basing on the number of N in the first HL. N was set to 5 difference values (8, 5, 4, 3 and 2, respectively) and the number of nodes in the second HL (when defined) was the same of the N of the first HL.

The maximum number of N (8) was estimated in according to previous study [42].

Table 2 shows the structure of the nine architectures used.

To verify the repeatability of our results we used a cross-validation scheme to train and test each ANN. All networks were trained 20 times by using a random 70% as the training test, a random 15% as validation set, and a random 15% as testing set. Accordingly, the prediction accuracy was computed as the average of 20 iterations.

A Confusion Matrix (CM) 2 × 2 was calculated for each ANN for 20 iterations and the mean of 20 CMs was provided for each network topology. A performance parameter (P) was calculated as the mean (%) of the elements on the diagonal of these CMs, where the 100% indicates the absence of misclassification. We selected the very architecture with the highest P (%) and the fastest computational speed.

## 3. Results

### 3.1. Clinical Results

Following the inclusion criteria, out of 30 patients extracted from our database, we included 21 patients in this study. Nine patients were excluded, due to incomplete EEG dataset, bilateral MR abnormalities or previous epilepsy surgery. Clinical, demographic and epileptological data of our patient cohort are shown in Table 3.

Among 21 patients, 15 (71%) were seizure and drug-free at last follow-up (Engel IA). Median follow-up duration was 5.1 years (range 2.3–12.6 years).

We observed a median age at surgery of 7 years (range 0.2–17.7 years).

Histopathological examination revealed: Rasmussen encephalitis in 4 cases (19%), malformations of cortical development in 5 (24%), unspecific gliosis/inflammatory infiltrates in 11 (52%), neuronal heterotopia in 1 (5%).

### 3.2. ANNs Performance

**Figure 3:**
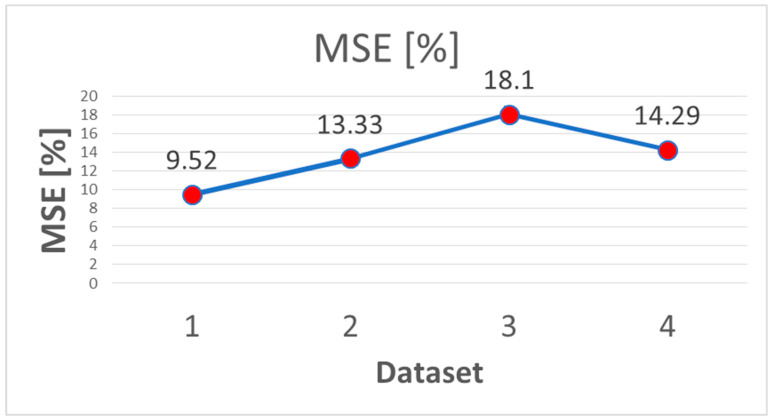
Experimental results using 4 different training dataset and comparison of recognition Mean Square Error (MSE).

As concerned features selection, we found that feature dataset 1 (all features) showed the lowest MSE (9.52%) in comparison to the other three training datasets (13.33%, 18.10% and 14.29% for the dataset 2, 3 and 4, respectively) (Figure 3).

Table 4 shows the prediction accuracy P (mean ± SD), with sensitivity and specificity (mean ± SD) values, related to the nine ANN architectures only for dataset 1, that turned out to be the best set of predictive feature for seizure outcome.

**Figure 4:**
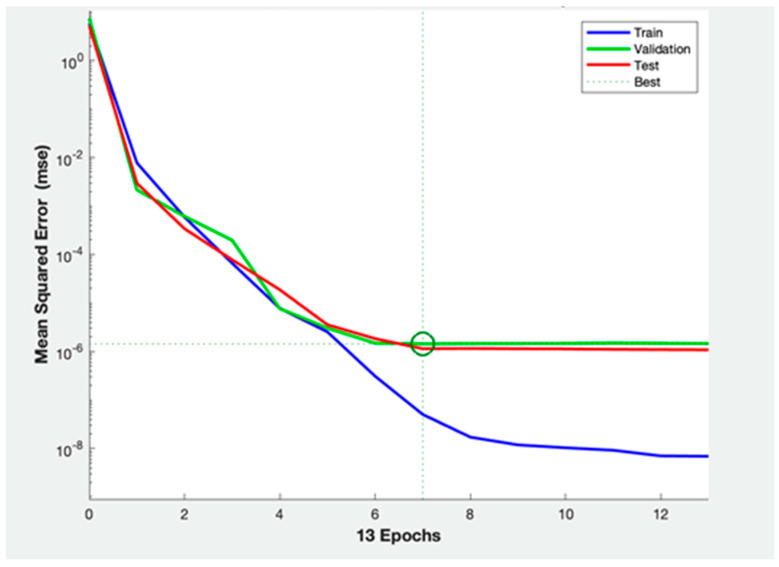
The Neural Network performance plot shows that overfitting and underfitting are irrelevant in our set. 1.4331 × 10^−6^ is the best performance value.

II ANN architecture resulted to have higher mean performance value with lower standard deviations (P = 73.33% ± 22.36%), compared with others. The “plotperform” shows that the II ANN architecture presents a good fit of EEG data and surgery outcome (Figure 4).

Table 5 shows the mean of CMs related to II architecture, showing that 85% of seizure free patients and 40% of non-seizure free patients were correctly classified.

## 4. Discussion

In this study, we evaluated, the efficacy of ML technology in investigating the correlation between preoperative inter-hemispheric brain connectivity and postoperative seizure outcome, in a series of pediatric patients who underwent hemispheric disconnections for drug-resistant epilepsy. To exclude possible bias related to bilateral pathology or surgical failure, we selected patients with electro-radiologic concordance and complete disconnection. Interhemispheric connectivity was measured with PSD: this analysis is the most used to extract EEG signal powers for each frequency band [43]. This method is influenced by the time-characteristics of the window-length analysis. It may affect the accuracy of the analysis of non-stationary signals [44]. For this purpose, we used also the Hjorth parameters to describe the characteristics of the EEG recording simultaneously in time and frequency domain [41]. The Hjorth analysis provides also a low computational cost [45].

Both radiologic [32,46] and intracranial neurophysiologic [47,48,49] ML-based studies demonstrated that altered brain connectivity may influence postsurgical seizure outcome. Neural network classifier examining DTI-based structural connectomes from 50 patients with temporal lobe epilepsy yielded a positive predictive value of 88% and negative predictive value of 79% in predicting Engel I outcomes [32]. Intracranial functional connectivity showed sensitivity and specificity higher than 85% in predicting seizure freedom, with a Support Vector Machine classifier [48,49].

Considering the homogeneous hemispheric involvement of our patients, we studied brain connectivity as inter-hemispheric comparison between presurgical EEG data [40,41], applying an automated ML-based method, with the hypothesis of a possible correlation between higher IC and worse postsurgical outcome.

IC in scalp EEG has been analyzed mainly in non-surgical contexts of epilepsy so far, such as generalized epilepsy, as childhood absence epilepsy, juvenile myoclonic epilepsy, photosensitive epilepsy syndromes [19,20,21,22], other neurologic conditions, like vascular injury [23] and other fields, as psychiatry [24,25,26]. IC has never been studied as a factor influencing seizure outcome after hemispheric surgery.

Over the last decade, growing studies reported ML-based prediction methods to address automated epilepsy diagnosis [28,29], seizure lateralization [30,31], and prediction of postsurgical seizure freedom [32,33,34,35,36]. These studies confirmed the key-role of ML techniques in uncovering prognostically valuable trends, on the basis of complex and multimodal data obtained during a typical presurgical evaluation, with aim of improving patient selection and counseling [50]. Moreover, ML was used to predict post-surgical outcome using unimodal input data, as clinical and neuropsychologic data [36], intracranial EEG [48,49], radiologic data [35], or multiple multimodal input data [33,36,51,52].

Despite the good accuracy in outcome prediction (usually higher than 80–90%) reported in these works, they involved both adult and pediatric patients and analyzed different surgical techniques, with strong limitation due to diffused overfitting.

In our study, we evaluated a whole pediatric cohort of patients, who underwent HT performed with the same surgical technique, through ML-based ANN approach.

Artificial Neuronal Network is an advanced ML technology, that develops learning patterns from large collections of data, by processing them through a multi-layer hierarchical architecture [53]. To our knowledge, brain machine learning approach was never attempted as a tool for predicting seizure outcome after hemispheric surgery and comparing our results with previous works might be difficult.

In our study, we adopted some selected frequency- and time-domain related EEG features as input data of ANN (Table 1). We subsequently combined these features, during the training phase, in four different datasets, with the purpose of minimize the number of parameters in the final ML model, improve performance and generalizability [54].

Dataset 1 (all the features) showed the lowest MSE with an accuracy level of 73.3%. Our model was effective in predicting seizure free patients (mean of 85%), while less powerful in detecting relapse cases (mean of 40%).

A recent review [55] examined ML performances applied to intracranial EEG, including 107 articles published from 2009 to 2020. ML techniques were used for different clinical purpose. Among papers dealing with seizure detection/prediction, sensitivity value reported ranged from 71% to 100%, specificity from 83.05% to 100%, and accuracy levels from 57.8% to 100%. These high levels of performance might be related to the type of signal, intracranial EEG, which has higher spatial resolution and lower noise of signal compared to scalp-EEG.

Previous studies reported even higher values of accuracy (more than 80–90%) in seizure outcome prediction, using the same ML classifier of our study (neural network) [51,52]. These series included adult patients, who underwent different epilepsy surgeries (anterior temporal lobectomy or lobar/multilobar resections, respectively) and trained their ANN with different data (clinical, electrographic, neuropsychologic, imaging, and surgical data). Multimodal data input were also used by other studies [33,36], with different ML classifiers, but the same high levels of accuracy (more than 89%) in predicting seizure outcome after temporal lobe surgery. It is conceivable that the use of multimodal variables of different nature, on a heterogeneous patient’s sample, might increase the prediction power of classifier, and this may in part explain the lower accuracy level that we obtained.

In our study, we obtained similar results if compared to previous published data. Previously cited papers had different data input, clinical sample, surgical population or ML classifier, therefore results cannot be matched together.

Compared to previous studies, we applied more selected inclusion criteria (homogeneous population with pediatric age, same clinical history, and surgery procedure) and decided to use only presurgical scalp EEG to train our neural network. This methodology allowed us to test a specific hypothesis of a possible relationship between presurgical IC and postsurgical outcome, regardless of any other variables.

## 5. Conclusions

The accuracy level of 73% that we obtained supports the hypothesis that presurgical EEG connectivity might have the potential to predict epilepsy outcome after HT.

Our study has several limitations. First, this is a retrospective analysis with a small sample of patients, compared with a high number of evaluated features, introducing overfitting. Moreover, we are also evaluating a scalp EEG signal, with lower spatial resolution in comparison with its invasive counterpart (stereotactic EEG or electrocorticography), higher level of noise and higher rate of interictal epileptiform abnormalities also in resting state. We obtained a mean accuracy level of 73%, from 20 iterations with random patient selection, therefore we do not predict single patient outcome or correlate outcome with specific etiology. We believe that the inclusion of different larger datasets may allow us to do single patient prediction and may overcome the problem of overfitting. The use of multimodal clinical/radiologic/psychological variables may also improve its accuracy [33].

Although relatively few cases were examined, our findings suggests that machine learning analysis might become a powerful tool to be included in standard evaluations for epilepsy surgery centers.

Our model’s accuracy of 73% does not allow for using it as a presurgical predictive tool in patients who will undergo hemispherotomy. Nevertheless, we believe that the implementation of our model with larger datasets may allow for generalizing our encouraging results in predicting outcome after surgery. Besides, this semi-automated tool does not require other examination than a routine video-EEG before surgery.

## Figures and Tables

**Table 1:** EEG features that we extracted and used for our ANN.

Type	Name	Description
Time Domain	Complexity	Hjorth parameters quantify the dynamical properties of signal
	Mobility	
	Activity	
Frequency Domain	Power Spectrum (PSD)	PSD quantify the signal power associated to specific frequency range

PSD: Power Spectrum Density.

**Table 2:** Nine different architectures of ANN (from I to IX) defined by a specific combination of Hidden Layers (HL) and number of Neurons (N).

ANNs	HL [1,2,3]	N	Architecture
I	1	8	8
II	2	8 × 2	8 + 8
III	3	5	5
IV	1	5 × 2	5 + 5
V	2	4	4
VI	3	4 × 2	4 + 4
VII	1	3	3
VIII	2	3 × 2	3 + 3
IX	3	2	2

**Table 3:** Clinical, demographic and epileptological data of our patient cohort. FCD: Focal Cortical Dysplasia; HME: Hemimegaloencephaly; MCD: Malformation of Cortical Development.

Patient	Age at Surgery	Etiology	Histology	Outcome	Follow-Up (Years)
1	1.3	HME	FCD Ic	IA	12.6
2	14.8	Rasmussen	Inflammatory infiltrate	IV	10.1
3	4.7	Perinatal ischemic injury	Gliosis	IA	4.8
4	17.7	Rasmussen	Gliosis	IA	7.6
5	2.9	HME	FCD IIa	IA	5.7
6	0.2	HME	FCD IIa	IA	5.8
7	4.5	Ischemic injury	Gliosis	IA	7.3
8	16.8	Vascular congenital abnormality	Gliosis	II	5.7
9	17.0	Perinatal ischemic injury	Gliosis	IV	7.5
10	7.0	Perinatal ischemic injury	Gliosis	IA	6.8
11	4.1	Hemispheric MCD	FCD Ic	III	4.4
12	0.2	HME	FCD IIa	IA	5.1
13	12.0	Post-infectious ischemic injury	Gliosis	IA	5.3
14	11.7	Perinatal ischemic injury	Gliosis	ID	4.9
15	7.0	Rasmussen	Inflammatory infiltrate	IA	4.8
16	8.8	Rasmussen	Inflammatory infiltrate	IA	2.3
17	17.9	Rasmussen	Inflammatory infiltrate	IA	2.9
18	0.7	Perinatal ischemic injury	Gliosis	IA	2.9
19	3.6	Rasmussen	Inflammatory infiltrate	ID	2.4
20	2.0	Hemispheric MCD	Neuronal heterotopia	IA	2.4
21	11.1	Rasmussen	Inflammatory infiltrate	IA	2.3

**Table 4:** This table shows the performance values of nine ANNs architectures. Highlighted in red, the one with the best classification performance (II) of 73.3 %.

Architecture	Accuracy (P%)	Specificity (%)	Sensitivity (%)
I	43.333 ± 14.91	61.000 ± 29.66	10.000 ± 22.36
II	73.333 ± 22.36	40.000 ± 54.77	40.000 ± 41.83
III	63.333 ± 29.81	86.667 ± 29.81	36.667 ± 41.50
IV	53.333 ± 18.26	83.333 ± 23.57	11.667 ± 16.24
V	66.667 ± 11.79	82.000 ± 20.49	20.000 ± 29.81
VI	63.333 ± 13.94	76.000 ± 43.36	25.000 ± 27.64
VII	63.333 ± 7.45	74.667 ± 25.56	40.000 ± 43.46
VIII	63.333 ± 24.72	63.333 ± 22.85	40.000 ± 54.77
IX	56.667 ± 9.13	65.667 ± 15.44	26.667 ± 43.46

**Table 5:** II architecture’s confusion matrix: the diagonal elements, highlighted in red, correspond to the number of correctly classified entries (True Positive = 85% and True Negative = 40%). The off-diagonal elements correspond to wrong classifications, False Negative and False Positive.

	Mean of Iteration		
ANNs		SF (%)	NSF (%)
II	SF	85	60
	NSF	15	40

## Data Availability

Data sharing not applicable.
